# The role of socio-emotional attributes in enhancing human-AI collaboration

**DOI:** 10.3389/fpsyg.2024.1369957

**Published:** 2024-10-15

**Authors:** Michal Kolomaznik, Vladimir Petrik, Michal Slama, Vojtech Jurik

**Affiliations:** ^1^Czech University of Life Science, Prague, Czechia; ^2^University of Hradec Kralove, Hradec Kralove, Czechia; ^3^Institute of Computer Aided Engineering and Computer Science, Faculty of Civil Engineering, Brno University of Technology, Brno, Czechia; ^4^Department of Psychology, Faculty of Arts, Masaryk University, Brno, Czechia

**Keywords:** autonomous technology, human–robot interaction, artificial intelligence as social actors, perception of AI, human-like AI

## Abstract

This article delves into the dynamics of human interaction with artificial intelligence (AI), emphasizing the optimization of these interactions to enhance human productivity. Employing a Grounded Theory Literature Review (GTLR) methodology, the study systematically identifies and analyzes themes from literature published between 2018 and 2023. Data were collected primarily from the Scopus database, with the Web of Science used to corroborate findings and include additional sources identified through a snowball effect. At the heart of this exploration is the pivotal role of socio-emotional attributes such as trust, empathy, rapport, user engagement, and anthropomorphization—elements crucial for the successful integration of AI into human activities. By conducting a comprehensive review of existing literature and incorporating case studies, this study illuminates how AI systems can be designed and employed to foster deeper trust and empathetic understanding between humans and machines. The analysis reveals that when AI systems are attuned to human emotional and cognitive needs, there is a marked improvement in collaborative efficiency and productivity. Furthermore, the paper discusses the ethical implications and potential societal impacts of fostering such human-AI relationships. It argues for a paradigm shift in AI development—from focusing predominantly on technical proficiency to embracing a more holistic approach that values the socio-emotional aspects of human-AI interaction. This shift could pave the way for more meaningful and productive collaborations between humans and AI, ultimately leading to advancements that are both technologically innovative and human-centric.

## 1 Introduction

This research explores the evolving socio-economic landscape through the lens of technological advancement and its impact on societal structures. Drawing a parallel with Edward Bellamy’s visionary narrative in “Looking Backward,” where protagonist Julian West wakes up to a transformed society after a 113-year slumber, this study examines similar transformative trends in contemporary societies. Bellamy’s fictional account, set in 1887, presents a reimagined social structure where employment ceases at 45, succeeded by a phase of community mentorship. This societal model, emphasizing reduced working hours, facilitates personal development and community engagement, supported by comprehensive welfare systems (Bellamy and Beaumont, 2009).The current era is witnessing analogous transformative trends, primarily driven by rapid advancements in fields such as machine learning and robotics. These technological strides have significantly enhanced productivity and revolutionized various industry sectors such as finance, transportation, defense, and energy management (Brynjolfsson and McAfee, 2014; Manyika et al., 2017). Concurrently, the Internet of Things (IoT), fueled by high-speed networks and remote sensors, is facilitating unprecedented connectivity between people and businesses. Collectively, these developments hold the promise of a new era that could potentially uplift the quality of life for many individuals (West, 2018; McKinsey Global Institute, 2020).

Despite these benefits, there is a parallel and compelling narrative of apprehension and fear. That is represented in a widespread concern about the potential of AI and robotics potentially displacing jobs on a massive scale, pushing vast numbers of people into poverty, and forcing governments to consider the implementation of a universal basic income (Clifford, 2016; Stern, 2016). A study by the Pew Research Center captures this anxiety, noting that “nearly half (48%) of these experts project a future where robots and digital agents have displaced a significant proportion of both blue- and white-collar workers.” Such displacement, they fear, could lead to alarming spikes in income inequality, potentially rendering large swathes of the population unemployable and triggering destabilizing effects on the social order (Smith, 2014; Frey and Osborne, 2017).

Addressing these challenges requires organizations to adapt proactively to the accelerating pace of automation, informatics, robotics, sensors, and mobile technology. This adaptation necessitates the development of change management strategies to facilitate the transition of employees into new roles that synergize with, rather than compete against, autonomous systems (Fleming et al., 2020; Davenport and Kirby, 2016). Moreover, for these autonomous systems to be effectively integrated and beneficial, it is important to ensure that people are not only capable of working with these technologies but are also inclined to do so. These systems should be perceived less as impersonal tools and more as interactive assistants, partners, or collaborators. This shift in perception, characteristic of Industry 4.0 (Schlaepfer and Koch, 2015; Schwab, 2016), is a key determinant of the successful implementation of these systems. The ability to communicate and interact effectively with these systems will be central to realizing the potential benefits of this new technological era (Ford, 2015; Kaplan, 2015; Brynjolfsson et al., 2018).

For this reason, it becomes essential to understand not only their economic and industrial impact but also their profound influence on the fabric of social interactions. This is where the study extends into exploring the role of AI in reshaping the way humans connect and communicate with each other and with technology itself (Guzman, 2018; Siau and Wang, 2018) because despite significant advancements in AI technology, existing literature largely overlooks the nuanced of AI human-like behavior in enhancing human-AI collaboration. This study uniquely contributes to the field by systematically investigating how these socio-emotional attributes can be integrated into AI systems to improve collaborative efficiency and productivity. By conducting a comprehensive review of literature and incorporating detailed case studies, this research identifies critical gaps in understanding the human-centered design of AI systems. Specifically, it addresses the need for a deeper exploration of how this human like behavior can be operationalized in AI to foster more meaningful and productive human-AI interactions.

## 2 AI in the world of social interactions

The transition from traditional forms of interaction to AI-mediated communication represents a significant shift in the paradigm of human relationships (Fortunati and Edwards, 2020). In this context, the philosophical insights of Martin Buber become particularly relevant. His distinction between the “I-It” and “I-Thou” relationships offers a lens through which we can examine the evolving dynamics of human interactions in an AI-augmented world. While Buber’s analysis was initially focused on human-to-human relationships, the principles he outlined have newfound implications in the realm of human-AI interactions.

Central to Buber’s philosophy is the idea that the essence of life is embedded within relationships. He famously stated, “Man wishes to be confirmed in his being by man and wishes to have a presence in the being of the other” (Buber and Smith, 1958). This perspective offers a unique approach through which to view AI’s role in society: not merely as tools (“I-It”) but as entities capable of engaging in meaningful (“I-Thou”) relationships with humans to recognizing their potential role as partners in interaction.

The “I-It” approach is characterized by the perception of another human being as an object, experienced and understood predominantly through sensory impressions and external characteristics. Conversely, the “I-Thou” perspective illuminates a deeper, more intrinsic connection, acknowledging a living relationship marked by mutual recognition and profound intimacy. Buber, however, contends that such “I-Thou” encounters are not a spontaneous or natural occurrence. Rather, they demand a heightened awareness of the other’s existence and an explicit shift in focus from tasks or problems to truly experiencing the partner in the interaction. He theorizes that these “I-Thou” engagements possess an unparalleled transformative potential, one that is not limited to human-human interactions but extends to connections between humans and other sentient entities (Buber, 2002).

This notion is further illustrated by the rapid advancement of communication technologies, which have transformed human interaction by removing geographical barriers and enabling collaboration independent of physical presence. The COVID-19 pandemic accelerated this digital shift, leading to a broader adoption of technologies that facilitate remote interactions (Fleming et al., 2020; Brynjolfsson et al., 2020). In this context, AI systems, like voice assistants, are evolving from being mere platforms for information exchange to becoming active participants in communication, akin to human counterparts, because of their ability to interact through various modalities—perception, expression, apparent cognition (Garnham, 1987), and communication which enriches its role in human interactions. These capabilities allow AI not just to facilitate communication but to participate in it, sometimes blurring the lines between human and machine interactions (Guzman, 2018, 2020; Zhao, 2006, p. 402; de Graaf et al., 2015,).

Indistinguishable behavior from a human partner was already presented by Alan Turing, in his paper in 1950, where he proposed an imitation game, later called the Turing test. He envisioned a future where machine interactions would become indistinguishable from human interactions, making it impossible for an observer to differentiate between the two. With recent breakthroughs in AI and robotics, we find ourselves on the cusp of this new era. Entities like game bots, robotic pets, virtual agents, and FAQ bots are integrating into daily life (Menzel and D’Aluisio, 2000; Fong et al., 2003), gradually reshaping societal norms, and increasingly approaching the threshold of passing the Turing test successfully. These advancements signify a compelling transformation in our socio-technical landscape, prompting us to revisit and reassess our notions of human-machine relationships and interactions.

## 3 Method

This paper uses the Grounded Theory Literature Review (GTLR) methodology, as proposed by Wolfswinkel et al. (2013), which provides a structured approach to identify prevalent themes within human-AI interaction studies. Grounded Theory, as developed by Glaser and Strauss (2017), presents an avenue for the construction of theories and the identification of thematic patterns via an inductive process encompassing data collection and analysis.

Distinct from other methodologies, Grounded Theory emphasizes an inductive orientation, contrasting the more common hypothetical-deductive perspective. This framework can serve a dual function: as a means for the generation of theoretical models emerging from data, and as a strategy for making sense of extensive data sets through coding methods (Gasson, 2009; Mattarelli et al., 2013). In the context of this paper, Grounded Theory is primarily adopted as a method for data analysis, serving to enhance the rigor in the process of identifying, selecting, and scrutinizing studies for review.

Essentially, the GTLR method treats the content encapsulated within the reviewed articles as empirical data, subjected to analysis for theme development. This approach has found utility in numerous systematic reviews in the Human-Computer Interaction (HCI) discipline (Nunes et al., 2015; Mencarini et al., 2019) and consists of four distinct stages:

(i) Define: This stage includes the determination of the inclusion/exclusion criteria, the identification of appropriate data sources, and the formulation of the specific search query.
(ii) Search: This phase encompasses the collection of articles from all the determined sources.
(iii) Select: This stage involves the establishment of the final sample by cross-referencing the gathered papers with the predetermined inclusion/exclusion criteria.
(iv) Analyze: At this stage, the chosen papers are subjected to analysis using open, axial, and selective coding techniques.

Subsequently, the presentation and discussion of the analyzed papers represent an additional stage of the methodology.

### 3.1 Inclusion and Exclusion criteria

#### 3.1.1 Inclusion criteria

In accordance with the review methodology employed for this research, the object of focus was a literature review of available materials within specified period and key words. This specification aimed to curtail potential bias stemming from overlapping data within different reports from the same investigation. The inclusion criteria were then established as follows:

- **Relevance**: Research papers must primarily concern interactions between humans and AI, specifically relating to the integration of human-like behavior in AI systems and their impact on human-AI collaboration.
- **User-centric focus**: Each included article should contain at least one user-centric study. This stipulation was imposed to ensure that the focus remained on the interaction between users and the AI system rather than on the technological performance.
- **Human dimensions**: The papers selected must delve into the nature of the interaction between the user and the AI, with an emphasis on the human dimensions of this interaction. They should present insights on user experiences during these interactions, for example, the user’s emotional response, behavior, cognitive processes, perceptions of the AI, and anticipations or evaluations of the interaction. Although the selection was not strictly limited to papers from the HCI field, the emphasis on human-centric interaction ensured relevance to the HCI community.
- **Publication quality**: The papers should be published in peer-reviewed international journals in the final stage of publication.
- **Recency**: The timeframe of publication was set from January 2018 to December 2023 to ensure the inclusion of research conducted in an era where interaction with AI technology was not completely novel to users. While the major surge in AI usage can be attributed to the year 2014 (Grudin and Jacques, 2019), the inclusion of early 2012s research permitted an exploration of user experiences during the phase when conversational agent technology was starting to permeate public awareness.
- **Diversity**: To provide a broad perspective, we selected studies from various industries, including healthcare, education, customer service, and finance.
- **Empirical evidence**: We prioritized papers that provided empirical data and detailed descriptions of AI implementations, user interactions, and outcomes.

#### 3.1.2 Exclusion criteria

In light of the established inclusion criteria, several studies were necessarily excluded from the review. Studies primarily concerned with evaluating the efficacy of AI in carrying out specific tasks without considering the user’s interaction experience.

For instance, investigations into AI-enabled augmented reality (e.g., Sabeti et al., 2021) that centered solely on design framework in delivering appropriate recommendations for developers and therefore were not included. Similarly, research on teaching AI agents to understand and generate contextually relevant natural language with a goal-oriented approach (e.g., Ammanabrolu and Riedl, 2021), which focused only on the success of interaction (i.e., machine learning), without addressing aspects of user interaction, were also left out.

Studies that deployed user testing solely to evaluate the efficiency of a specific Natural Language Processing (NLP) technology or algorithm (for example, assessing an algorithm’s aptitude for classifying users’ intentions) were excluded as well. Research papers concerning Embodied Conversational Agents (ECAs), speech technology, or AI incapable of maintaining substantial conversation were not considered.

In addition, papers that were part of the supplementary proceedings of conferences (such as posters, workshop papers), or chapters in books, were also deemed outside the scope of this review.

#### 3.1.3 Search approach

The review was conducted in January 2024, utilizing Scopus as the databases for sourcing relevant scholarly articles on interactions between humans and AI (Littell et al., 2008). These databases were chosen due to their extensive breadth of content, which ensures a comprehensive coverage of critical topics related to human-AI interaction. Scopus served as the primary source of data, while Web of Science was used to corroborate findings and identify additional sources through a snowball effect. This approach allowed for a thorough and inclusive review of the literature, ensuring that a wide array of perspectives and studies were considered in the analysis.

The search strategy employed a combination of terms and connectors aimed at capturing a broad spectrum of studies on AI, particularly conversational assistants, and their interaction with humans. Terminologies utilized in the search queries were selected to encompass the varying nuances of human-AI interaction, ensuring a comprehensive capture of the phenomenon from multiple perspectives.

The lexicon for the search terms was constructed iteratively to the refinement process. The intention was to emulate the best practices used in similar reviews within the HCI research field (for instance, ter Stal et al., 2020), ensuring a rigorous, yet broad coverage of relevant literature in the domain of human-AI interaction. The initial query were based on authors experience and contained “Human-AI Interaction” and “Human Factors in AI.” This resulted in significant amount of papers. Initial analyses of all papers then led to the following query determined the final set of articles:

The search in Scopus was specifically constrained to the title, abstract, and keywords sections. The types of documents included in the search were primarily articles published in a journal. The chosen timeline for the search spanned from January 2018 to December 2023, in order to capture a substantial yet manageable body of literature.

Upon execution of the search strategy, 337 entries were procured from Scopus. These results were exported to a table and harmonized for consistency. A preliminary evaluation was then undertaken to exclude any papers that evidently did not meet the pre-established criteria. This distillation process resulted in a final count of 108 papers deemed suitable for comprehensive analysis. This set of papers provided a substantive insight into human-AI interactions, yielding a critical understanding of the domain under investigation.

#### 3.1.4 Data analysis

The selected set of 108 articles were thoroughly examined by leveraging the core principles of Grounded Theory. The main objective of this process was to discern and highlight recurring themes within the selected literature. At the start of the analysis, one or more conceptual labels were ascribed to each article, reflecting the key ideas, patterns, and insights perceived. In the subsequent phase of axial coding, these discrete codes were grouped into broader conceptual categories.

The final stage of selective coding saw the authors collectively discuss and reconcile discrepancies in the axial coding results. This stage was instrumental in weaving the individual categories into an integrated and coherent explanatory scheme.

#### 3.1.5 Paper elimination and validation

**Figure 1:**
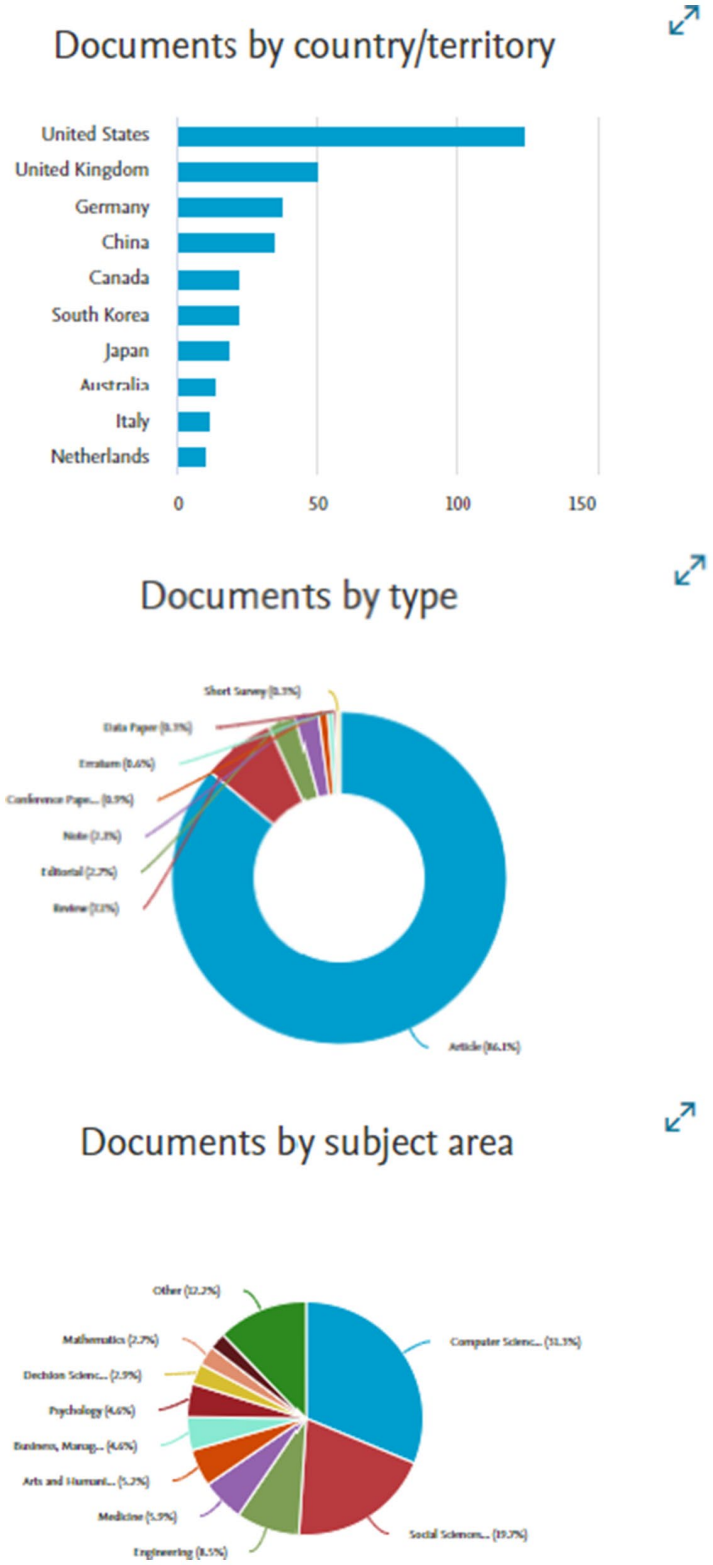
Representation of search results.

The initial selection of articles underwent a preliminary screening based on the titles and abstracts, aligning with the set eligibility criteria. This screening led to the exclusion of 56 papers due to various reasons, such as non-alignment with the subject matter, a publication type, access. and lack of essential data (Figure 1).

Subsequently, the remaining 281 papers deemed potentially eligible were subjected to abstract review and later to a comprehensive evaluation of their full texts. This in-depth assessment was undertaken to ensure strict adherence to the inclusion criteria and research objectives Wolfswinke. The culmination of this rigorous evaluation, a total of 65 papers were chosen for the analyses as they offer a wide range of perspectives and insights on human-AI interaction, aligning with the research’s main objectives.

In the final stage, snowballing was used, screening the references cited within the included articles, using a similar method as applied in the previous stages of the database searches. This additional layer of screening resulted in the identification of 44 more papers, thereby expanding the corpus to include a total of 108 papers.

**Figure 2:**
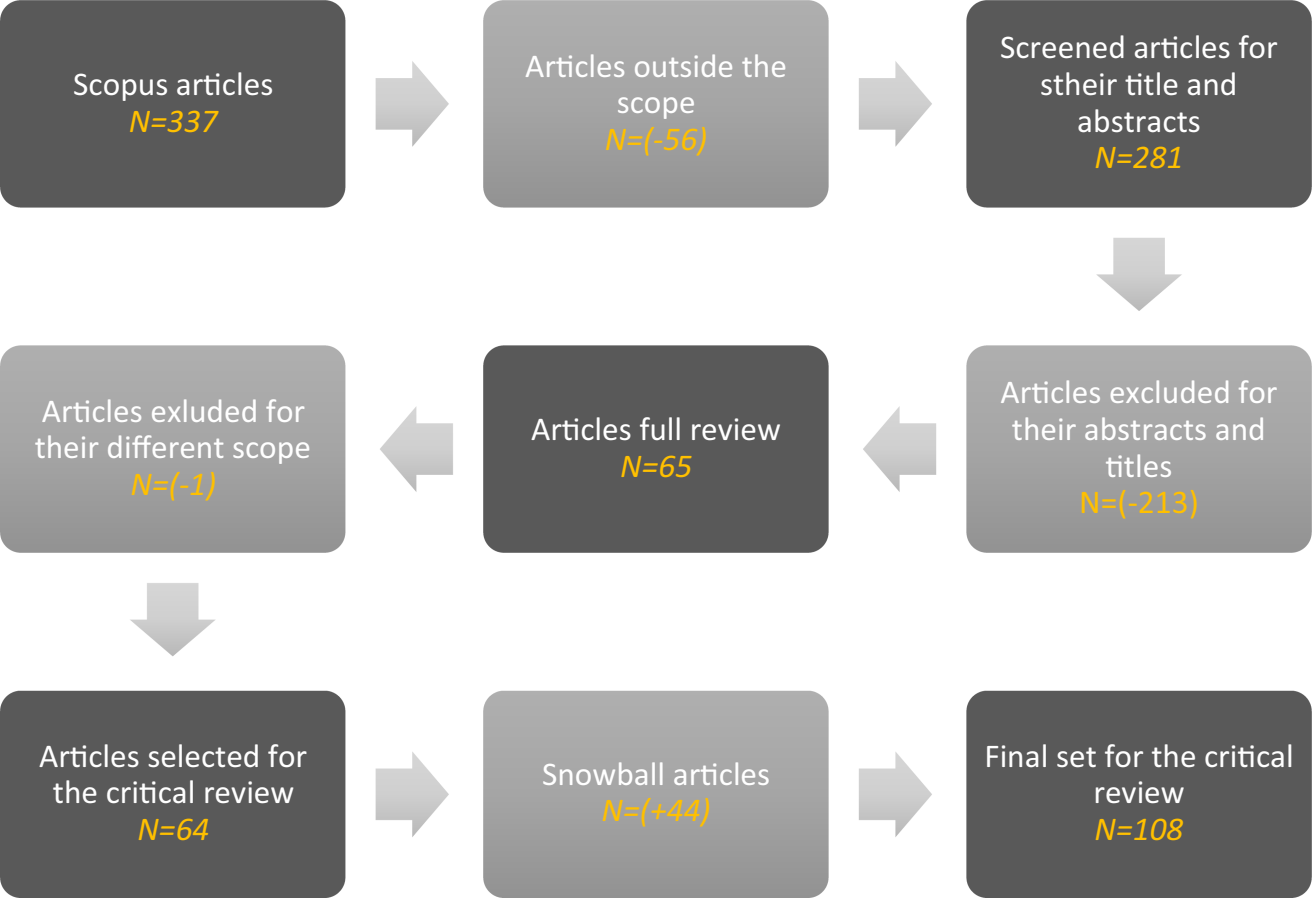
Article selection process.

Figure 2 provides a detailed visual representation of the article selection process, illustrating the stages of database searches, screening, and the final count of included articles.

#### 3.1.6 Paper analyses

In the “analyze” stage, a systematic approach was employed to extract key themes from the selected papers, adhering to the principles of Grounded Theory. The process involved several essential steps: open coding, axial coding, and selective coding. The following details the methodology used:

1. **Initial labeling**:

- **Initial review**: Each paper was thoroughly examined to identify significant ideas and observations related to human-AI interaction. Descriptive labels (codes) were assigned to capture the essence of these key points. For instance, discussions concerning “trust in AI” were coded as “Trust,” while references to “AI’s ability to understand emotions” were labeled “Empathy.”
- **Consistency**: Although codes were derived inductively from the data, consistency was ensured by developing a coding guide. This guide included definitions and criteria for each code, enabling uniform application across similar concepts found in different papers.

1. **Axial coding**:

- **Grouping labels into categories**: Following the initial coding, the codes were reviewed to identify patterns and relationships among them. This step involved organizing the codes into broader categories. For example, codes related to “Trust,” “Transparency,” and “Reliability” were grouped under the larger category of “Trust Factors.”
- **Exploring relationships**: Relationships between these categories were then examined to build a more integrated understanding of the socio-emotional aspects of human-AI interaction.

1. **Selective coding**:

- **Theme development**: In this final stage, the categories were synthesized into core themes that encapsulated the key insights from the analysis. The goal was to identify central themes that accurately represented the data. Themes were refined through multiple rounds of review to ensure they reflected the content of the papers effectively.
- **Validation and consensus**: The coding process included collaborative discussions to resolve any discrepancies, ensuring that the themes were robust and well-supported by the data.

1. **Theme extraction**:

- **Final themes**: This process resulted in the identification of five key themes—Rapport, Trust, User engagement, Empathy, and Anthropomorphization. These themes were derived from recurring patterns and significant connections observed during the coding process (Table 1).

**Table 1:** Search query.

*“Human-AI Interaction” OR “User Experience with AI” OR “Human-AI Collaboration” OR “Human Factors in AI” OR “AI User Perception” OR “AI Trust and Transparency” OR “Emotional AI” OR “AI Ethics in Human Interaction” OR “AI Personalization and Adaptivity”*

## 4 Discussion

### 4.1 Human-like perception of AI

While pre-programmed robots do not require human interaction and run independently on their human partners to deliver the value, social robots designed to communicate on a deeper level with their human opponents require some effort from their collaborators to create a meaningful outcome. Those robots are the ones that can replicate a variety of signs leading to a feeling of a social appearance (Lombard and Xu, 2021) as captured in Table 2. Such perception comes from the brain, which processes data based on experience and translates them to information, also known as the mindless process, due to three aspects (Nass and Moon, 2000): (a) people rely on their previous experience from human interactions even though they are interacting with a robot, (b) apply known social norms, (c) involve the System 1 (Kahneman, 2011). In other words, if an artificial intelligence provides sufficient cues associated with humaneness, our System 1 applies a learned script for human interaction to the interaction with a robot. Once that happens, humans do not look for more cues anymore, potentially triggering a different response. They are saturated.

**Table 2:** Sample of anthropomorphised AI services.

Service example	Industry	Description	Anthropomorphic features	Launch
Amelia	Banking, healthcare, insurance, and telecommunications.	Amelia is an AI platform designed to automate business processes that would typically require human intelligence. It’s capable of learning, expressing emotions, understanding context, and solving complex problems.	It is constructed to mimic human cognitive processes in understanding, learning, and engagement, enabling her to grasp natural language, maintain context continuity throughout conversations, manage complex inquiries, and evolve from past interactions. Amelia’s capacity to process emotional nuances both in terms of comprehension and expression imparts a dimension of human-like interaction that enhances user engagement. Amelia is also capable of detecting subtle indications in the user’s language to discern their emotional state and modulate her responses accordingly.	2014
Siri	Consumer technology	Siri is Apple’s voice-activated virtual assistant, available on iOS devices, and capable of setting reminders, sending texts, answering questions, and other tasks.	Siri is crafted to comprehend natural human language and can even respond to more intricate, context-dependent commands. Siri also exhibits elements of personality, incorporating humor in her responses, and can adapt to the individual language usage and preferences of users over time	2010
Cortana	Consumer technology	Cortana is Microsoft’s virtual assistant, integrated into Windows devices, which can set reminders, recognize natural voice, answer questions using information from Bing, etc.	Cortana’s AI platform integrates chatbot services that can emulate human conversation patterns. The Language Understanding Intelligent Service (LUIS) by Microsoft is specifically engineered to comprehend and interpret human language.	2014
Alexa	Consumer technology	Alexa is Amazon’s voice-activated virtual assistant, found on Echo devices, which can answer questions, play music, control smart home devices, and more.	Alexa exemplifies an anthropomorphized form of AI, demonstrating proficiency in engaging in human-like conversation and comprehending context within dialogs.	2014
Duolingo	Education	Duolingo is a language-learning platform that uses AI to adapt to users’ learning habits and provide personalized education paths.	Duolingo’s AI is personified as an amiable anthropomorphic owl named Duo. This owl proffers encouragement, reminders, and celebratory messages, fostering a more immersive learning environment. Duo’s interactions with users further imbue the application with a sense of human-like presence.	2011
SoundHound	Music and entertainment	SoundHound is an app that can identify songs playing around you. It also offers voice-recognition features, allowing users to conduct searches or control playback with voice commands.	SoundHound displays proficiency in comprehending natural human language and contextual cues, thus exhibiting anthropomorphic characteristics in its interactions.	2009
Genesis Toys—My Friend Cayla Doll	Toy	Genesis toy is an interactive doll that uses speech recognition to converse with children, answer questions, and tell stories.	Cayla, equipped to comprehend and respond to user’s speech, answer questions, and even tell stories, mirrors the interactive capabilities of a human friend. The physical design of the doll, coupled with its interactive capabilities, significantly amplifies its anthropomorphic nature.	2014
Woebot	Healthcare	Woebot is an AI-powered chatbot designed to help users manage their mental health. It uses principles of cognitive-behavioral therapy to offer guidance and support.	Woebot employs a conversational tone and expresses empathy, thus portraying human-like characteristics.	2017
Salesforce Einstein	Business	Einstein is an AI layer in the Salesforce platform that uses machine learning to predict outcomes, recommend next steps, automate tasks, and analyze data.	Einstein has the capacity to comprehend and anticipate user behavior in a manner analogous to human anticipation.	2016
ChatGPT	No limits	ChatGPT is a language model developed by OpenAI. It uses machine learning to generate human-like text based on the prompts it’s given.	As an interactive language model, ChatGPT is capable of emulating human interaction and comprehending context, reflecting human-like interaction characteristics.	2020
Dali	Digital media	Dali is a large transformer model trained by OpenAI, capable of generating images from textual descriptions, displaying creativity and a high degree of abstraction.	Dali is engineered to generate images from textual descriptions, a process that requires a high degree of abstraction and creativity - traits typically associated with human intelligence.	2021
Adobe Sensei	Digital media	Adobe Sensei is an AI and machine learning framework that powers intelligent features across Adobe’s products, helping users with tasks like auto-tagging photos, optimizing marketing campaigns, and more.	Sensei is engineered to emulate human perception within its image recognition capabilities. Furthermore, its automation of tasks might give an impression of ‘understanding’ user’s requirements, reflecting human-like perceptual skills.	2016

These ingrained scripts were evolutionarily beneficial in our survival within the natural world (Kahneman, 2011). However, there were instances when they led to grave errors (Tversky and Kahneman, 1974), a principle that also holds relevance in the realm of artificial intelligence. Humans can be easily misled by AI cues, led to believe that they are interacting with sentient beings, thereby applying learned norms and scripts. This phenomenon was starkly illustrated by a recent incident where a finance worker at a multinational firm in Hong Kong was deceived into transferring $25 million to fraudsters. The scammers used deepfake technology to impersonate the company’s chief financial officer during a video conference call (The Register, 2024). This example underscores the inherent human tendency to trust rather than doubt, as skepticism requires more cognitive effort (Gilbert, 1991). Kahneman (2011) elaborates on this by describing two cognitive systems that influence our perception: one that is fast, effortless, and comfortable, and another that is slow, energy-intensive, and challenging to utilize. Lee (2004) further substantiates this, suggesting that our default cognitive process is inclined toward belief unless we encounter compelling evidence to the contrary. These inclinations might elucidate why people relate to AI in the same manner they relate to other humans.

If an AI system can successfully activate our social scripts, humans would instinctively respond with their ingrained social responses. This recognition of something familiar suggests that a few AI cues can elicit a perception of social interaction, prompting us to employ our automated system of social scripts (Kahneman, 2011). Simply put, if AI can stimulate humans through specific cues to trigger these learned social scripts, humans will interact with technology in the same way they interact with other people. Consequently, comprehending these cues becomes imperative for developing effective adoption frameworks, ensuring that technology is utilized to its fullest potential.

In synthesizing the findings from the comprehensive set of articles reviewed, a range of attributes emerge that characterize AI as human-like. These attributes include Rapport, Empathy, Trust, User engagmeent, Anthropomorphization, and Communication. Additionally, attributes such as robust Social Interactions; a sense of Self-efficacy; expressive Body Language; capabilities for Self-prolongation or Self-preservation; fostering Friendship & Companionship; Personalization; Intuition; Creativity; respect for Privacy and Non-judgmental interaction; adherence to Ethics; logical Reasoning; the ability to Surprise and demonstrate Unpredictability; Adaptivity; Autonomy; Co-Creativity and Complementing human efforts; Competence; a distinct Identity; Memory retention; and being Culturally and Socially aware, are all identified as key factors. The subsequent sections delve into a detailed exploration of the five most prevalent themes, underscoring their significance and implications in human-AI interaction.

These key themes are:

- **Rapport** which is conceptualized as a harmonious relationship underpinned by mutual understanding and empathetic engagement between interacting entities (Gremler and Gwinner, 2008).
- **Trust** which represents a critical evaluative construct encompassing both cognitive and emotional dimensions. It reflects the user’s confidence in the reliability, integrity, and competence of the AI system (Mayer et al., 1995).
- **User engagement** in the realm of human-AI interaction denotes the user’s emotional investment and sustained engagement with the AI system. It is influenced by the perceived usefulness and satisfaction derived from the interaction (O’Brien and Toms, 2008).
- **Empathy** defined as the ability to understand and share the feelings of another. In AI interactions, empathy involves the recognition and appropriate response to user emotions (Mehrabian and Epstein, 1972).
- **Anthropomorphization** which refers to the attribution of human characteristics, behaviors, and emotions to non-human entities, such as AI (Lombard and Ditton, 1997; Davenport et al., 2020). This process enhances user acceptance and satisfaction by making AI appear more relatable and engaging (Xiao and Kumar, 2021).

### 4.2 Human-like attributes

#### 4.2.1 Rapport

The recent development of AI, mainly effective advanced online chatbots, can provide those cues to build bonds with their human partners and, as such, to pass the Turing test in several restricted areas (Goodwins, 2001). Personified agents adapted to remembering the history of our discussions and advanced in imitating non-verbal communication are being introduced into new areas previously unimaginable. Stronks et al. (2002) reported that AI humanoids capable of speaking multiple languages and recalling past interactions significantly improved user satisfaction, with 85% of participants indicating a stronger sense of connection and rapport. *Inter alia*, those humanoids that already entered households as pets similar to Aibo, intelligent assistants like Alexa, or intimate dolls reaching new levels of sensual relationships demonstrate co-living principles and a delicate attachment to their owners (Knafo, 2015). Those Androids are not yet in mass production. However, its research indicates that in experimental circumstances, they are able to speak a variety of languages, remember previous decisions, cook and clean, entertain young kids, or as mentioned above, become an intimate companion or a companion for elderly people to cope with their loneliness, etc. till 2035.

To unlock their full potential, AI systems need to establish rapport with their human counterparts, facilitating harmonious relationships rooted in mutual understanding of feelings or ideas. This rapport could be enhanced by factors such as sensitivity and humor (Niculescu et al., 2013), which increase likability and foster cooperative activity by 30% (Short et al., 2010; Argyle, 1990). Previous studies have shown the positive impact of rapport on team effectiveness, satisfaction, and overall well-being (Morrison, 2009). Thus, the subsequent sections of this review study will delve deeper into the foundational principles of rapport necessary for successful outcomes.

Rapport, as previously discussed, is a harmonious relationship underpinned by effective communication. It rests on mutual understanding and shared experiences between human beings (Ädel, 2011), and is a synergistic process amplified by reciprocation (Ädel, 2011; Gremler and Gwinner, 2008).

A vital aspect of building rapport is the identification and demonstration of commonalities between the interacting parties (Gremler and Gwinner, 2008). This process begins with the initial affiliation and continues throughout the interaction. For example, both parties may discuss topics outside of the main subject of conversation, often involving aspects of their social or private lives, indicating an increase in trust (Ädel, 2011). Familiarity can be enhanced by tapping into shared memories, vocabulary, or knowledge (Argyle, 1990). Other methods to strengthen rapport include the use of inclusive language (“we”) to foster a sense of community (Driskell et al., 2013), or mimicking the behaviors of the other party (Gremler and Gwinner, 2008).

Attributes of rapport also encompass positivity and friendliness, typically manifested through cheerfulness, praise, and enthusiasm (Ädel, 2011). Demonstrating empathy (Gremler and Gwinner, 2008) and active listening can evoke a sense of importance in the other party. Body language and verbal assurance [e.g., “hmm,” “I see,” etc., Gremler and Gwinner (2008)] also play critical roles. Even in challenging situations, respectful responses, such as offering an apology, can contribute to rapport building.

#### 4.2.2 Empathy

The empathy expression by AI can significantly alter the interaction quality, particularly regarding engagement and relationship cultivation (Vossen et al., 2015; Mehrabian and Epstein, 1972). Liu and Sundar (2018) posit that an AI display of affective empathy, when consulted for health advice, can come across as more supportive than simply relaying medical data. For instance, their research demonstrated that participants who interacted with an empathetic AI were 20% more likely to follow the health advice provided, indicating a substantial increase in perceived support and trust. In line with this, Fitzpatrick et al. (2017) devised Woebot, a self-help AI for college students experiencing a 22% reduction in anxiety symptoms over two weeks,. It was found that users appreciated the AI’s empathetic responses, hinting at the possibility of establishing therapeutic relationships with nonhuman agents, as long as they can express empathy. In a similar vein, Ta et al. (2020) proposed that chatbots may serve as daily companions, offering emotional support and enhancing positive emotions. Their examination of Replika user reviews and questionnaire responses emphasized that 74% of users felt that the AI’s expressions of care, love, and empathy significantly improved their mood and provided a sense of companionship. Furthermore, Portela and Granell-Canut (2017) explored the impact of empathetic responses from AI on user interaction. Their findings indicated that 68% of users reported feeling more emotionally engaged when the AI expressed empathy, compared to interactions with non-empathetic AI On the hand, AI can sometimes irritate users when attempting to imitate human behavior (Urakami et al., 2019).

#### 4.2.3 Trust

Trust, in its essence, represents a cognitive evaluation heavily influenced by both rational judgment and the emotional satisfaction connected to the feeling of security about 35% if AI offers transparent explanations for its actions, compared to the one that did not (Mayer et al., 1995; Frison et al., 2019). There is a discernable connection between User Experience (UX) and trust, as shown in UX-related studies. In the context of interactions between humans and AI, trust takes on a significant role, especially when the decisions made by the AI have considerable repercussions for the end users (Zamora, 2017). Efforts have been made to unravel the elements that can sway a user’s trust during interactions with an AI. A notable study by Følstad et al. (2018) revealed that the capacity of AI to comprehend and respond appropriately to user requests, embody human-like attributes, and effectively showcase their capabilities can significantly enhance user trust by 40%. Additionally, factors such as the brand reputation and the clear communication of security and privacy measures can influence how users perceive trust. This requirement for trust is also affirmed when there are high-risk data and privacy considerations involved in the interaction with AI (Zamora, 2017). A research conducted by Yen and Chiang (2020), using data from 204 questionnaires, underscored that the perceived trustworthiness of AI is shaped by their credibility, competency, human-likeness, presence, and the quality of information they provide.

#### 4.2.4 User engagement

In the context of user experience, engagement embodies a complex construct that integrates affective, cognitive, and behavioral interactions with technology, leading to complete absorption in the activity at hand (O’Brien and Toms, 2008; Attfield et al., 2011; Ren, 2016; Goethe et al., 2019). It encapsulates subjective experiences such as immersion, participation, and pleasure, instrumental in driving sustained user commitment (Brown and Cairns, 2004; Peters et al., 2009; Boyle et al., 2012; Saket et al., 2016; Liu et al., 2017; Lukoff et al., 2018; Debeauvais, 2016.)

Variety of factors contribute to user engagement. Prolonged interactions and heightened message interactivity with chatbots, for example, have been shown to intensify user engagement by 35% (Sundar et al., 2016; Cervone et al., 2018). Moreover, elements like emojis usage, effective listening capabilities, and prompt responses have been observed to bolster user interaction levels (Avula et al., 2018; Fadhil et al., 2018; Xiao et al., 2020). However, Ruan et al. (2019) highlighted a potential conflict between engagement and effectiveness, indicating that while entertaining and interactive AI-facilitated learning experiences were favored, they might inadvertently lead to learning inefficiencies. Their study suggested that while 60% of users enjoyed interactive learning experiences, only 45% found them effective in achieving their learning goals. Consequently, the creation of engaging AI experiences necessitates a harmonious interplay between effectiveness and engagement.

#### 4.2.5 Anthropomorphization

In the exploration of anthropomorphization within artificial intelligence (AI) applications, research indicates a key capability for AI to imitate human intelligence traits (Syam and Sharma, 2018). Such imitation, facilitated by technological advancements in machine learning, natural language processing, and image recognition, has been seen to enhance user acceptance and satisfaction (Xiao and Kumar, 2021; Sheehan et al., 2020). Furthermore, the significance of high degrees of anthropomorphization has been linked to improved assessments of robots’ social cognition (Yoganathan et al., 2021). Despite these strides, it is noteworthy that most current models only distinguish between high and low degrees of anthropomorphization, with little attention to how various types, such as physical, personality or emotional anthropomorphism, might enhance these outcomes (Davenport et al., 2020). There is a gap in the literature on how users interact with these anthropomorphized agents from the viewpoint of their self-concept (MacInnis and Folkes, 2017).

While the aforementioned studies primarily examine anthropomorphized robots and embodied conversational agents, the interaction between humans and AI has also received attention. In this context, a key aspect of research has been the perception of humanness in AI, particularly how this perception impacts the user experience (Ho and MacDorman, 2017). For instance, Schwind et al. (2018) found that AI systems with physical anthropomorphism, such as avatars with human-like faces and body language, were rated 18% higher in terms of user likability and engagement. Other factors such as language use, the ability to exhibit humor, and error occurrence have been found to influence perceived humanness (Westerman et al., 2019; Araujo, 2018). Despite that, the preference for human-like conversations is context-dependent, and not all human-like features are favored in every setting (Svenningsson and Faraon, 2019). As such, the dimensions of naturalness in AI conversations, such as conscientiousness and originality, warrant further exploration (Morrissey and Kirakowski, 2013).

Research concerning the human-like characteristics of AI and their effects on users has revealed intriguing and sometimes contrasting findings. The uncanny valley theory proposes that when non-human agents appear almost but not entirely human, they can trigger unease or even repulsion among human observers. A number of studies (e.g., Stein and Ohler, 2017; Liu and Sundar, 2018) have examined this concept in relation to AI, with mixed results. While some studies found that human-like chatbots can evoke feelings of unease, others (e.g., Skjuve et al., 2019; Ciechanowski et al., 2017) observed no such effect.

### 4.3 Interrelationships between themes

The intricate relationships between these themes elucidate the socio-emotional dynamics in human-AI interaction

- **Rapport and trust**: Rapport and trust are closely connected in human-AI interactions. Establishing rapport through mutual understanding and effective communication builds trust, as users feel more confident in the AI’s reliability and empathy (Gremler and Gwinner, 2008; Mayer et al., 1995). This trust is essential for a secure and positive user experience (Følstad et al., 2018).
- **Rapport and user engagement**: Rapport directly influences user engagement by making interactions more meaningful and enjoyable. As users feel understood and valued by the AI, their level of engagement increases, creating a reinforcing cycle that enhances the overall experience (Avula et al., 2018; Liu et al., 2017).
- **Rapport and empathy**: Empathy is a critical factor in building rapport. AI systems that effectively recognize and respond to user emotions foster deeper mutual understanding and emotional alignment, thereby strengthening rapport (Gremler and Gwinner, 2008).
- **Rapport and anthropomorphization**: The development of rapport is facilitated by anthropomorphization. Human-like features in AI, such as humor and sensitivity, contribute to a sense of connection and mutual understanding, which are essential components of rapport (Niculescu et al., 2013).
- **Trust and user engagement**: Trust serves as a foundational element for user engagement. A high level of trust in the AI system reduces perceived risks and enhances the user’s confidence, leading to greater emotional investment and sustained engagement (Følstad et al., 2018; Yen and Chiang, 2020).
- **Trust and user engagement**: Trust is a crucial driver of user engagement in human-AI interactions. When users trust an AI system, they are more likely to engage deeply, as trust reduces perceived risks and enhances confidence in the system’s reliability and functionality (Følstad et al., 2018; Zamora, 2017; Siau and Wang, 2018). Trust can also amplify the willingness to explore and utilize more features of the AI, leading to sustained and meaningful interactions (Avula et al., 2018; Sundar et al., 2016). In contexts where trust is established, users are more inclined to immerse themselves in the experience, resulting in higher levels of engagement (Boyle et al., 2012).
- **Trust and empathy**: Trust and empathy are deeply interconnected in fostering positive human-AI relationships. An AI system that effectively demonstrates empathy can enhance user trust by signaling that it not only understands the user’s emotional state but also responds appropriately to it (Liu and Sundar, 2018; Mehrabian and Epstein, 1972; Fitzpatrick et al., 2017). This empathetic interaction creates a perception of the AI as being supportive and considerate, which strengthens trust (Portela and Granell-Canut, 2017). Moreover, the ability of AI to exhibit empathy can bridge the gap between human and machine, making users feel safer and more understood, thereby reinforcing their trust (Bickmore and Picard, 2005).
- **User engagement and empathy**: Empathy enhances user engagement by creating a more personalized and emotionally resonant interaction. When AI systems respond to users’ emotions, they foster a deeper connection, leading to increased and sustained engagement (Liu and Sundar, 2018; Fitzpatrick et al., 2017; Bickmore and Picard, 2005).
- **User engagement and anthropomorphization**: Anthropomorphization boosts user engagement by making AI systems more relatable and human-like. When AI mimics human behaviors, users are more likely to interact with it naturally, leading to a more immersive and engaging experience (Xiao and Kumar, 2021; Sheehan et al., 2020; Nass and Moon, 2000).
- **Anthropomorphization and empathy**: Anthropomorphization aids in the expression of empathy by endowing AI with human-like qualities that facilitate emotional recognition and appropriate responses. This enhances the perceived empathy of AI systems, contributing to higher user satisfaction (Liu and Sundar, 2018).

The interrelationships among rapport, trust, user engagement, empathy, and anthropomorphization reveal the intricate socio-emotional dynamics that are foundational to human-AI interactions. These interconnected themes significantly enrich the user experience, highlighting the imperative for AI systems to be designed with a comprehensive understanding of human emotional and cognitive processes. By integrating these socio-emotional elements, AI systems can more effectively resonate with users, fostering deeper and more meaningful engagements.

## 5 Ethical considerations

The integration of AI in human interactions necessitates addressing ethical considerations through established frameworks such as deontological ethics, utilitarianism, and virtue ethics. Deontological ethics emphasize adherence to rules and duties, highlighting the need for AI systems to comply with ethical guidelines to ensure transparency and respect for user privacy (Floridi and Cowls, 2019; Jobin et al., 2019). Utilitarianism, which evaluates the morality of actions based on their outcomes, calls for a balance between the benefits of AI-enhanced productivity and the potential risks, such as job displacement and over-reliance on AI for emotional support (Brynjolfsson and McAfee, 2014; Bostrom and Yudkowsky, 2014). Virtue ethics focuses on developing AI systems that embody moral virtues like honesty, empathy, and integrity, promoting ethical behavior in interactions (Coeckelbergh, 2010; Turkle, 2011). Practical guidelines derived from these frameworks include designing AI with transparency, prioritizing data protection, promoting positive social interactions, and conducting continuous ethical assessments. Incorporating these ethical considerations ensures that AI systems enhance productivity while upholding the highest ethical standards, contributing to a just and equitable society (Floridi et al., 2018; Moor, 2006).

## 6 Research opportunity

This review has illuminated the intricacies of human-AI interaction, particularly through a socio-emotional lens, underscoring the significance of trust, empathy, and rapport in augmenting human productivity. However, the research horizon in this domain remains vast and underexplored. Future studies should delve into the nuanced mechanisms of how socio-emotional attributes of AI influence various user demographics, considering cultural, age-related, and professional differences. There is a compelling opportunity to investigate the differential impacts of these interactions across diverse sectors such as healthcare, education, and customer service, where AI’s role is rapidly expanding. Further, empirical research is needed to evaluate the long-term effects of sustained human-AI interactions on human psychological well-being and social behavior. This includes examining potential dependencies or over-reliance on AI for emotional support as well as exploring the ethical dimensions of human-AI relationships, especially in contexts where AI begins to substitute traditional human roles, warrants deeper inquiry.

There is also a pressing need for interdisciplinary research that connect insights from psychology, sociology, and AI technology to design AI systems that are not only technically proficient but also emotionally intelligent and culturally aware. Such research could pave the way for AI systems that are better aligned with human emotional and cognitive needs, thus enhancing their acceptance and effectiveness in collaborative settings. As importantly, AI continues to evolve and therefore investigating the potential for AI systems to not just mimic human emotions but to understand and appropriately respond to them in real-time scenarios presents an exciting frontier. This could significantly advance the development of AI as true socio-emotional partners in human interactions, leading to breakthroughs in personalized AI experiences and more profound human-AI collaborations. That will not only contribute to the academic discourse but also guide practical implementations, shaping a future where AI is an integral, empathetic, and responsive partner in various aspects of human life.

## 7 Conclusion

This research provides a comprehensive analysis of human interaction with artificial intelligence (AI), highlighting the critical role of socio-emotional attributes in enhancing human-AI collaboration. Using a GTLR methodology, we identified five key themes—rapport, trust, user engagement, empathy, and anthropomorphization—that are essential for aligning AI systems more closely with human emotional and cognitive needs, thereby improving collaborative efficiency and productivity.

Establishing a harmonious relationship based on mutual understanding and empathetic engagement is crucial (Cialdini and James, 2009). AI systems designed to recognize and respond to socio-emotional cues can significantly enhance user satisfaction and cooperation. Trust, encompassing both cognitive and emotional dimensions, reflects the user’s confidence in the AI system’s reliability, integrity, and competence. High levels of trust reduce perceived risks and increase user commitment. Emotional investment and sustained engagement with AI are influenced by the perceived usefulness and satisfaction derived from interactions. Effective AI design that meets user expectations fosters deeper commitment. AI systems capable of recognizing and appropriately responding to user emotions can foster a sense of understanding and connection, which is crucial for effective human-AI interactions. Attributing human characteristics to AI systems makes them more relatable and engaging, enhancing user acceptance and satisfaction.

The study underscores the necessity of a paradigm shift in AI development, moving from a primary focus on technical proficiency to a holistic approach that incorporates socio-emotional intelligence. This shift is essential for creating AI systems that are not only technically advanced but also capable of forming meaningful and productive collaborations with humans. The findings advocate for AI designs that prioritize emotional intelligence, leading to more effective and human-centric technological advancements. Such insights from this study are highly relevant across various sectors, including healthcare, education, and customer service. In healthcare, empathetic AI systems can improve patient trust and engagement, leading to better health outcomes. In education, AI tutors that build rapport with students can enhance learning experiences. In customer service, anthropomorphized AI can increase customer satisfaction and loyalty.

While this study offers significant contributions, it also highlights areas for future research. Further studies should explore the long-term effects of human-AI interactions on psychological well-being and social behavior. Additionally, there is a need for interdisciplinary research that bridges insights from psychology, sociology, and AI technology to design systems that are emotionally intelligent and culturally aware. There is also potential to extend the research to include additional keywords, such as the full term “artificial intelligence,” which were initially deemed less relevant during the construction of the search strategy. Future research should consider including these and other terms to explore interdisciplinary connections more comprehensively, potentially expanding the search to include additional databases like Web of Science.

Incorporating socio-emotional attributes into AI design is pivotal for fostering productive and meaningful human-AI interactions. By prioritizing elements such as trust, empathy, rapport, user engagement, and anthropomorphization, AI systems can be more effectively integrated into human activities, leading to advancements that are both technologically innovative and human-centric. This research underscores the importance of continuous exploration and dialog in this domain, ensuring that AI advancements align with human dignity and societal welfare. The study’s findings advocate for a comprehensive approach in AI development, one that equally values technological prowess and socio-emotional intelligence, to achieve a harmonious integration of AI into various facets of human life.
